# Anaphylaxis-induced premature uterine contractions: a case report and literature review

**DOI:** 10.1186/s12884-024-06297-2

**Published:** 2024-03-13

**Authors:** Puntabut Warintaksa, Waranyu Lertrat, Roberto Romero, Pornpun Vivithanaporn, Paninee Mongkolsuk, Threebhorn Kamlungkuea, Rapeewan Settacomkul, Pisut Pongchaikul, Piya Chaemsaithong

**Affiliations:** 1 Department of Obstetrics and Gynecology, Faculty of Medicine, Ramathibodi Hospital, Mahidol University, Bangkok, 10400 Thailand; 2 Pregnancy Research Branch, Division of Obstetrics and Maternal-Fetal Medicine, Division of Intramural Research, Eunice Kennedy Shriver National Institute of Child Health and Human Development, National Institutes of Health, U.S, Department of Health and Human Services (NICHD/NIH/DHHS), Bethesda, MD 20892 USA; 3 Department of Obstetrics and Gynecology, University of Michigan, Ann Arbor, MI 48109 USA; 4 Department of Epidemiology and Biostatistics, Michigan State University, East Lansing, MI 48824 USA; 5 Chakri Naruebodindra Medical Institute, Faculty of Medicine Ramathibodi Hospital Mahidol University, Samut Prakan, 10540 Thailand; 6 Integrative Computational BioScience Center, Mahidol University, Nakhon Pathom, 73170 Thailand; 7 Institute of Infection, Veterinary and Ecological Sciences, University of Liverpool, Liverpool, L69 3BX UK

**Keywords:** Allergy, Amniotic fluid, Anaphylaxis, Contractility, Contraction, Hypersensitivity, Myometrial contraction, Pregnancy, Preterm, Uterine allergy

## Abstract

**Background:**

Preterm labor is caused by multiple etiologies, including intra-amniotic infection and/or intra-amniotic inflammation, vascular disorders, cervical disease, decidual senescence, and breakdown of maternal–fetal tolerance. Accumulating evidence *in vivo* and *in vitro* has shown that an allergic reaction, including anaphylaxis, can induce preterm uterine contractions. This report describes a case of a pregnant woman who developed anaphylaxis and regular uterine contractions after the ingestion of a strawberry-coated biscuit. We also review the mechanism of allergic reaction (hypersensitivity)-induced preterm labor.

Case presentation

A 31-year-old woman (gravida 1, para 0) at 30^+2^ weeks of gestation was admitted to the labor and delivery unit with regular uterine contractions and anaphylactic symptoms after she ingested a strawberry-coated biscuit as a snack. The uterine contractions resolved after the treatment of anaphylaxis by administering antihistamines and epinephrine. The patient subsequently delivered at 39^+3^ weeks of gestation. The amniotic fluid profile showed no infection or inflammation. A postpartum skin-prick test confirmed a positive type 1 hypersensitivity reaction to the strawberry-coated biscuit.

**Conclusions:**

We report a case of anaphylaxis-induced uterine contractility in which uterine contractions subsided after the treatment of anaphylaxis. The absence of intra-amniotic infection and/or intra-amniotic inflammation and the cause of the anaphylaxis were confirmed. Our findings indicate that maternal allergic reactions may be one of the mechanisms of preterm labor.

## Background

Preterm birth is the leading cause of neonatal mortality globally [1–7]. Multiple pathological mechanisms, such as intra-amniotic inflammation, intra-amniotic infection, vascular disorders, cervical disease, decidual senescence, and breakdown of maternal–fetal tolerance, lead to spontaneous preterm delivery [1, 8–11]. Among these mechanisms, intra-amniotic infection and/or intra-amniotic inflammation is causally linked to preterm delivery [10, 12–17]. Accumulating evidence has shown that a maternal allergic reaction, including anaphylaxis, can induce preterm uterine contractions, which resolve after treatment of the allergic reaction, and the patient subsequently delivers at term gestation [18, 19]. The mechanism responsible for uterine contractions is thought to be myometrial contractility induced by degranulation of mast cells (effector cells of type 1 hypersensitivity) [18]. Anaphylaxis is a rare complication of pregnancy with an incidence rate ranging from 1.6 to 2.7/100,000 deliveries [20–23]. Common causes of anaphylaxis during pregnancy include the use of antibiotics and food allergies [21, 23]. This condition is associated with maternal hypotension and hypoxemia, which are potentially life-threatening to the mother and fetus [23]. Anaphylaxis is initially managed by immediate improvement of the maternal airway, eliminating causative agents, and administering drugs, such as anti-histamines, epinephrine, glucocorticoids, and vasopressors [19–23]. These drugs can be used safely without major side effects in pregnancy.

We report a case of a pregnant woman who developed systemic anaphylaxis and regular uterine contractions after ingestion of a strawberry-coated biscuit. Her uterine contractions subsided after the administration of antihistamines and epinephrine without the administration of tocolytic agents. Subsequently, she delivered uneventfully at term (39^+3^ weeks’ gestation). No intra-amniotic infection or intra-amniotic inflammation was associated with the uterine contractions. The cause of anaphylaxis was confirmed at postpartum. We also discuss the mechanism of anaphylaxis implicated in preterm labor.

## Case presentation

**Fig. 1:**
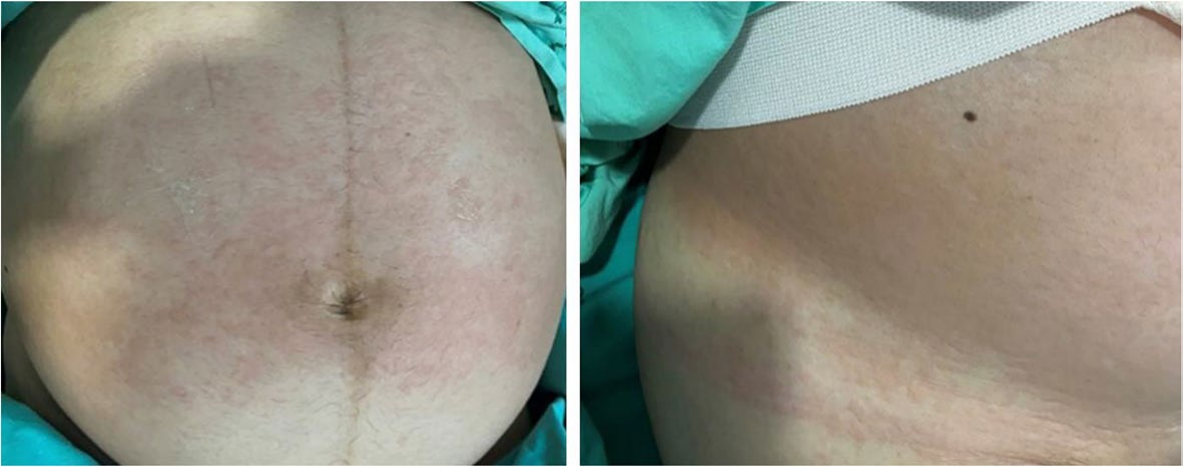
Urticarial rash on the patient’s abdomen. Photo courtesy of Dr. Piya Chaemsaithong, with permission

A 31-year-old gravida 1, para 0, Thai woman at 30^+2^ weeks of gestation presented to the labor and delivery unit with regular uterine contractions together with an acute onset of generalized hives, pruritus, flushing, and swollen lips. Her antenatal care had been unremarkable. Prior to this pregnancy, she had several episodes of urticaria, but she had never investigated the cause of the rash. Six hours before hospital admission, she developed nasal and throat itching, tightness in the chest, shortness of breath, nausea, and vomiting. She also developed swollen lips and tongue, and an itchy, urticarial rash that began at her face and trunk and radiated to the upper and lower extremities (Fig. 1). These symptoms occurred suddenly after snacking on a strawberry-coated biscuit. She also reported an episode of regular abdominal cramping every 3 minutes. Her physician administered chlorpheniramine 10 mg intravenously and referred the patient to our hospital because of the presentation of preterm uterine contractions.

**Fig. 2:**
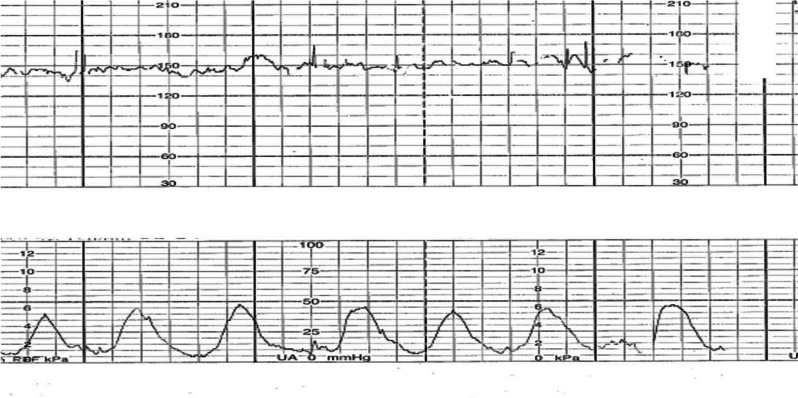
External fetal monitoring. Uterine contractions occurred every 3 minutes and 40 seconds with moderate intensity

At the labor and delivery unit (6 hours after the onset of symptoms), the patient complained of shortness of breath and abdominal cramps. Her vital signs showed tachycardia with a heart rate of 120 beats/minute and tachypnea with a respiratory rate of 24 breaths/minute. Her blood pressure was 100/60 mmHg. At a physical examination, inspiratory and expiratory wheezing in both lungs was observed. Facial swelling, erythema, edema, and a generalized pruritic erythematous maculopapular rash at the chest wall and abdomen and over both extremities were also observed (Fig. 1). The size of her uterus was appropriate for gestational age with the presence of regular uterine contractions occurring every 3 minutes and 40 seconds with moderate intensity (Fig. 2). A digital examination showed a closed cervical os with no effacement. The fetal heart rate status was reassuring, as shown by a baseline fetal heart rate of 140 beats/minute. An ultrasonographic examination revealed a single fetus appropriate for gestational age, the amniotic fluid and placenta appeared normal, and the patient’s cervical length was 30.2 mm.

**Fig. 3:**
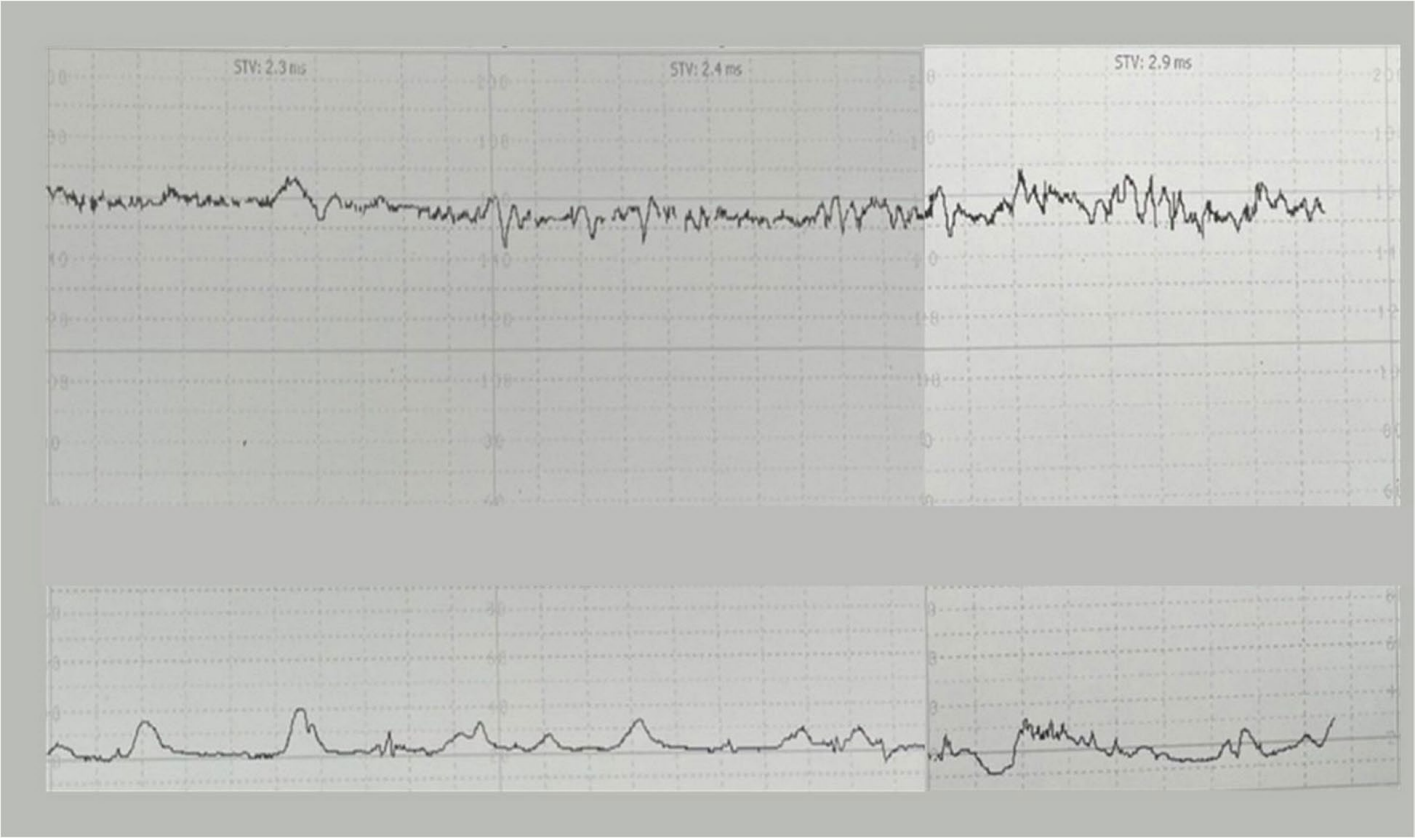
Resolved uterine contractions after antihistamine and epinephrine administration

The patient was diagnosed with anaphylaxis and threatened preterm labor. To treat her anaphylaxis, intravenous chlorpheniramine 10 mg, intramuscular epinephrine (1:1000) 0.5 mg, and intravenous dexamethasone 5 mg were administered. Two hours after the administration of epinephrine, the patient still had uterine contractions. A digital examination was performed and the cervical os was closed with 50% effacement. Therefore, preterm labor was diagnosed. Preterm labor syndrome is associated with intra-amniotic inflammation and/or intra-amniotic infection. Therefore, the patient was counseled about amniocentesis (under the IRB approval protocol COA. MURA2021/254). Written informed consent was obtained from the patient before collecting specimens. Second doses of intramuscular epinephrine (1:1000) 0.5 mg and intravenous dexamethasone 5 mg were administered to remedy the persistent dyspnea and wheezing. Her dyspnea and uterine contractions improved within 1 hour after administering the second doses of epinephrine and dexamethasone (Fig. 3). The maculopapular rash and swelling subsided within a few days.

At the admission (6 hours after the onset of symptoms), her maternal white blood cell (WBC) count was 16,580 cells/mm^3^ (neutrophils: 84.2%, lymphocytes: 5.9%, and monocytes: 6.9%). An amniotic fluid analysis showed a WBC count of 105 cells/mm^3^ (polymorphonuclear cells: 46%, mononuclear cells: 54%). Hematoxylin and eosin staining for a cell differential was not performed. Amniotic fluid Gram staining, culture, and 16S ribosomal RNA gene polymerase chain reaction results were negative for microorganisms, and the amniotic fluid interleukin-6 concentration was within the normal range (0.41 ng/mL; cut-off > 2.6 ng/mL). These negative findings were consistent with no finding of intra-amniotic infection or intra-amniotic inflammation. The pregnancy progressed uneventfully, and the patient subsequently had a vaginal delivery of a female neonate weighing 3,480 g at 39^+3^ weeks of gestation. The neonate’s Apgar score was 9, 10 and had no complication.

Three months after the delivery, the patient experienced similar symptoms (i.e., tightness in the chest and a maculopapular rash) after ingesting another snack of the same brand. The cause of the allergy was investigated. A skin-prick test result at 6 months after the delivery showed a positive wheal and flare for a strawberry-coated biscuit.

## Discussion

This report suggests that maternal anaphylaxis can induce the onset of preterm uterine contractions, and these can be resolved by the administration of antihistamine agents, adrenergic drugs, and steroids. Although several reports have described the association between an allergic reaction and uterine contractions, only a few investigations proved the cause of allergy [18, 19]. Additionally, none of these reports determined the status of intra-amniotic infection and/or intra-amniotic inflammation, which is frequently observed in women presenting with symptoms of preterm labor.

## Anaphylaxis during pregnancy

Anaphylaxis occurring during pregnancy is a rare event with an incidence of approximately 3.8/100,000 hospitalizations [20–22]. The mortality rate of anaphylaxis is approximately 0.09/100,000 live births [23]. According to diagnostic criteria amended by the World Allergy Organization in 2020 [24], anaphylaxis is a clinical diagnosis based on the criteria shown in Table 1.

**Table 1:** Diagnostic criteria of anaphylaxis [24]

**Criteria 1**	Acute onset of an illness (minutes to several hours) with simultaneous involvement of the skin, mucosal tissue, or both (e.g., generalized hives, pruritus or flushing, swollen lips-tongue-uvula)	
	and at least one of the following:	a. Respiratory compromise (e.g., dyspnea, wheeze-bronchospasm, stridor, reduced peak expiratory flow, hypoxemia)
		b. Reduced blood pressure or associated symptoms of end-organ dysfunction (e.g., hypotonia, syncope, incontinence)
		c. Severe gastrointestinal symptoms (e.g., severe crampy abdominal pain, repetitive vomiting), especially after exposure to non-food allergens
**Criteria 2**	Acute onset of hypotension or bronchospasm or laryngeal involvement after exposure to a known or highly probable allergen (minutes to several hours), even in the absence of typical skin involvement	

A recent systematic review and meta-analysis of 47 pregnant women with anaphylaxis showed that the most common clinical manifestations are hypotension and tachycardia (100%), followed by urticaria (57%) [22]. In addition, anaphylaxis is more commonly observed during the period of labor (approximately 80%) than prior to the onset of labor. Antibiotics, anesthetic drugs, latex, oxytocin, misoprostol, and rubber contained in a Foley catheter are common allergens. Anaphylaxis can be associated with a 3.2% (95% confidence interval: 0.4–11) maternal mortality rate and a 14.3% (95% confidence interval: 4.8–30.3) neonatal encephalopathy rate [21]. Nevertheless, approximately half of the reported cases were solely diagnosed based on clinical symptoms and allergological tests without a proven allergen [22]. In the current case, the criteria of anaphylaxis were met. Therefore, we concluded that the anaphylaxis induced preterm uterine contractions. Indeed, uterine contractions are recognized as a manifestation of anaphylaxis and, importantly, the cause of anaphylaxis can be proven after delivery.

## Allergy as a mechanism of disease of preterm labor

Evidence supportive of an allergic reaction (hypersensitivity) as one of the mechanisms implicated in preterm labor has been reported as follows.

1. In 1910, Schultz reported that the exposure of a specific allergen to a sensitized guinea pig induced contractions of the ileum [25]. Subsequently, a contractile response was demonstrated in the uterus of the sensitized guinea pig [26]. The “Schultz-Dale phenomenon” was then characterized by a standard technique in which antigen-induced contractions of the smooth muscles *in vitro* induced anaphylactic hypersensitivity [27]. Schultz-Dale anaphylactic contractions have been shown in the uterus of the guinea pig [28–46], rat [44, 47–49], mouse [50, 51], and human [52]. The Schultz-Dale experiment was later demonstrated in human myometrial strips collected from women with an allergy to ragweed. In addition, a higher frequency and intensity of myometrial contractions were observed in pregnant donors and then compared with non-pregnant donors [53]. Moreover, preincubation of myometrial strips in human serum led to the sensitization of the myometrial strips of non-allergic women, and this was mediated by immunoglobulin E [53].
2. Mast cells, which are the effector cells of an allergic reaction, have been found in the uterus [54]. Furthermore, histamine and prostaglandins produced by mast cell degranulation can induce myometrial contractility [54, 55].
3. Pharmacological degranulation of mast cells with a compound called “48/80” induces myometrial contractility [56–58]. Histamine and serotonin induce myometrial contractions in a dose-dependent manner [53, 54, 59, 60]. Diphenhydramine, chlorpheniramine, and antihistamine agents partially suppress myometrial contractility of sensitized myometrium strips challenged with an allergen, suggesting a histamine receptor-mediated mechanism [61, 62]. In addition, pre-treatment with cromolyn, which is a mast cell stabilizer, inhibits myometrial contractility, suggesting a role of mast cells, especially degranulation, in myometrial contractions [61, 62].
4. Incubation of myometrial strips from sensitized and non-sensitized rats with an anti- immunoglobulin E antibody increases myometrial contractility [56].
5. A challenge with ovalbumin results in an increase in the uterine tone of non-pregnant guinea pigs sensitized with ovalbumin [56–58].
6. The autopsy report of a guinea pig, performed immediately after its death caused by anaphylactic shock, showed increased uterine contractility [25].
7. Eosinophils identified in the amniotic fluid were found to represent the majority of WBCs in a subset of women with preterm labor [63]. Eosinophil-rich inflammation is associated with allergic diseases, such as asthma, atopic dermatitis (eczema), and allergic rhinitis [64–68]. Additionally, patients with an increase in amniotic fluid eosinophils (WBC count differential containing > 20% of eosinophils) are at an increased risk of preterm delivery. However, some patients with eosinophils detected in samples of amniotic fluid deliver at term and show no evidence of complications [63].
8. Preterm labor and delivery can be induced in a guinea pig model of type I hypersensitivity [18]. In addition, a study showed that pretreatment with a histamine H1 receptor antagonist (ketotifen) to ovalbumin-sensitized guinea pigs increased the duration of gestation and prevented preterm labor and delivery [61].
9. Previous studies showed that guinea pigs that were sensitized with chicken egg ovalbumin were at greater risk to deliver a preterm pup than those challenged with normal saline [18, 61].
10. Prior studies have suggested an association between patients with an allergic disease, such as asthma or allergic rhinitis, and increased preterm delivery [69–71].
11. Several case reports have shown that exposure to an allergen can induce uterine contractions and that these contractions are ameliorated after the treatment of anaphylaxis together with the standard treatment of preterm labor pain [22, 72–74]. Collectively, type I hypersensitivity reactions can induce preterm labor and delivery.

## Type I hypersensitivity reactions and uterine contractions in humans

A systematic review of clinical studies reported 31 cases of uterine contractions induced by anaphylaxis [19]. A total of 29% (9/31) of these cases were in pregnant women, of whom seven of nine of these women presented with an allergic reaction and preterm uterine contractions. Three cases were anaphylactic [73, 75]. Table 2 shows reported cases of type I hypersensitivity-induced preterm uterine contractions.

**Table 2:** Summary of cases with type I hypersensitivity-induced premature uterine contractions

**Number**	**Authors/Year**	**Patient**	**Details of reaction**	**Presentation**	**Treatments**	**Response to treatment**	**Final outcome**
1.	Klein et al. 1984 [72]	32 year-old, 29 weeks of gestation	Reaction following the ingestion of crab and cherries	Pruritus and urticaria, palmoplantar erythema, hypotension and uterine contractions. Fetal heart rate 138 bpm then 150 bpm with repetitive decelerations.	Diphenhydramine 50 mg, IV fluids, oxygen, ephedrine 5 mg IV ephedrine 10 mg IV	The contractions continued despite the treatment, the deceleration diminished gradually within 25 minutes with complete resolution after 2 hours.	Cesarean delivery at 40^+5^ weeks of gestation. The baby was normal.
2	Habek et al. 2000 [73]	23 year-old 27 weeks of gestation	Reaction following by a wasp sting	Swollen face, eyelids, lip, tongue. Tachypnea, dyspnea, bronchospasm, uterine contractions	Adrenaline 0.5 mg for 3 doses, IV fluids, oxygen, Methylprednisolone 500 mg IV, Aminophylline 250 mg IV, 10% calcium gluconate 10 ml IV	Facial edema and uterine contractions subsided Normal cardiotocography examination	Delivery at 35 weeks of gestation
3	Donahue et al. 1995 [76]	26 year-old 19 weeks of gestation	Telangiectasia macularis eruptiva perstans (rare type of mastocytosis)	Chest tightness, dyspnea, uterine contractions, vaginal bleeding and maculopapular rash	Chlorpheniramine, Terbutaline aerosols, Promethazine	Dysnea and uterine contractions relieved after treatment	Preterm labor at 24 weeks treated with magnesium sulfate. A normal baby was born by Caesarean delivery at 36 weeks
4	Kehoe et al. 2006 [77]	35-year-old 24 weeks of gestation	Systematic mastocytosis Anxiety and stress	Uterine cramping, peptic ulcer disease, diarrhea,flushing, fatigue, and rash	H1 and H2 blocker, proton pump inhibitor, leukotriene inhibitors	Relieved uterine contractions and other symptoms	Spontaneous delivery at term gestation with 3,310g female infant
5	Madendag et al. 2010 [78]	26-year-old 27 weeks of gestation	Cutaneous mastocytosis	Cutaneous manifestations with pruritus and premature uterine contractions	Pheniramine maleate 50 mg IV	Cutaneous manifestations and uterine contractions were resolved	Spontaneous vaginal delivery at 40 weeks of gestation
6	Romero et al. 2010 [18]	28 year-old 31 weeks of gestation	Symptoms occurred shortly after ingestion of lobster	Generalized pruritic maculopapular rash, regular strong uterine contractions every three minutes	Chlorphenamine and betamethasone orally	Rash and uterine contractions subsided	Cesarean delivery at 40 weeks of gestation and delivered infant weighed 3800 g. The child developed atopic disorders.
7	Tsuzuki et al. 2017 [75]	26 year-old 25 weeks of gestation	Symptoms occurred 15-30 min after meal	Bronchospasm with peripheral cyanosis, generalized itchy rash, abdominal pain and regular uterine contractions	Oxygen Epinephrine 0.4 mg intramuscula Nebulized short-acting β2-receptor agonist	Bronchospasm, rash and uterine contractions resolved	Delivery at 37 weeks of gestation. Skin prick test revealed buckwheat allergy.

The first case of uterine contractions induced by hypersensitivity or anaphylaxis during pregnancy was reported in 1984 [72]. A pregnant woman at 29 weeks of gestation developed erythema of the palms and urticarial areas on the face and abdomen, uterine contractions every 3 to 4 minutes, and maternal hypotension. Fetal heart rate tracing showed repetitive late decelerations. After the administration of diphenhydramine and ephedrine, the uterine contractions and the late decelerations of fetal heart rate were resolved. The patient underwent cesarean delivery at term because of a failure to progress in labor. The newborn had an Apgar score of 9-9, a birthweight of 3780 g, and a normal neurological exam [72]. The second case was an anaphylactic reaction in response to a wasp sting in a woman at 27 weeks of gestation, followed by preterm delivery at 35 weeks of gestation [73]. Three patients were diagnosed with mastocytosis, which is characterized by an abnormal accumulation of mast cells in several organs. These patients experienced pruritic urticaria pigmentosa and preterm uterine contractions at mid-gestation, which were resolved by the administration of intravenous pheniramine maleate, hydroxyzine hydrochloride, and imipramine hydrochloride. All patients eventually delivered at near term or at term gestation without complications [76–78].

Subsequently, Romero *et al* described a pregnant woman at 31 weeks of gestation with an episode of spontaneous preterm labor and a generalized pruritic maculopapular rash after the ingestion of shellfish [18]. Preterm labor subsided after the treatment of antihistamines and steroids. The patient eventually delivered at 40 weeks of gestation. Recently, Tsuzuki *et al* reported a pregnant woman at 25 weeks of gestation who presented with anaphylactic symptoms (i.e., dyspnea, generalized itchy rash, and regular uterine contractions) after consuming buckwheat noodles. Such symptoms subsided after the administration of epinephrine, antihistamine, and methylprednisolone. A healthy, neurologically intact neonate was delivered at term. Skin-prick and challenge test results of the mother confirmed a buckwheat allergy [75].

In this report, we describe the fourth case of pregnancy with anaphylaxis-induced preterm uterine contractions. Anaphylaxis is generally diagnosed based on clinical criteria, which were met in this patient (acute onset of skin and laryngeal involvement), even though tryptase concentrations were not measured in the samples of serum and amniotic fluid. Strengths of this report are the confirmation of the cause of the allergic status post-delivery and the finding that the patient’s uterine contractions were not associated with intra-amniotic inflammation or intra-amniotic infection. Notably, an analysis of the types of cells in amniotic fluid by a flow cytometer or hematoxylin and eosin staining would be useful because a high number of eosinophils might be associated with type 1 hypersensitivity, including anaphylaxis, which can induce preterm labor [63].

## Conclusions

We report a case of anaphylaxis-induced uterine contractions for which treatment of the anaphylaxis was followed by the resolution of uterine contractions. This evidence highlights a maternal allergic reaction as one of the mechanisms of preterm labor.

## Data Availability

All data generated or analyzed for this case report are included in this published article.

## Abbreviation

WBC: White blood cell

## References

[1] Goldenberg RL, Culhane JF, Iams JD, Romero R (2008). Epidemiology and causes of preterm birth. Lancet.

[2] Iams JD, Romero R, Culhane JF, Goldenberg RL (2008). Primary, secondary, and tertiary interventions to reduce the morbidity and mortality of preterm birth. Lancet.

[3] Beck S, Wojdyla D, Say L, Betran AP, Merialdi M, Requejo JH (2010). The worldwide incidence of preterm birth: a systematic review of maternal mortality and morbidity. Bull World Health Organ.

[4] Chawanpaiboon S, Vogel JP, Moller AB, Lumbiganon P, Petzold M, Hogan D (2019). Global, regional, and national estimates of levels of preterm birth in 2014: a systematic review and modelling analysis. Lancet Glob Health..

[5] Lee AC, Blencowe H, Lawn JE (2019). Small babies, big numbers: global estimates of preterm birth. Lancet Glob Health.

[6] Chawla D, Agarwal R (2022). Preterm births and deaths: from counting to classification. Lancet Glob Health.

[7] Dhaded SM, Saleem S, Goudar SS, Tikmani SS, Hwang K, Guruprasad G (2022). The causes of preterm neonatal deaths in India and Pakistan (PURPOSe): a prospective cohort study. Lancet Glob Health.

[8] Romero R, Espinoza J, Kusanovic JP, Gotsch F, Hassan S, Erez O (2006). The preterm parturition syndrome. BJOG.

[9] Romero R (2009). Prenatal medicine: the child is the father of the man. 1996. J Matern Fetal Neonatal Med..

[10] Romero R, Dey SK, Fisher SJ (2014). Preterm labor: one syndrome, many causes. Science.

[11] Jung E, Romero R, Yeo L, Chaemsaithong P, Gomez-Lopez N. Intra-amniotic infection/inflammation and the fetal inflammatory response syndrome. In: Polin R, Abman SH, Rowitch DH, Benitz W, editors. Fetal and neonatal physiology, 6th ed. Elsevier: Amsterdam, Netherlands; 2022.

[12] Schultes V, Deutzmann R, Jaenicke R (1990). Complete amino-acid sequence of glyceraldehyde-3-phosphate dehydrogenase from the hyperthermophilic eubacterium Thermotoga maritima. Eur J Biochem.

[13] Gomez R, Romero R, Edwin SS, David C (1997). Pathogenesis of preterm labor and preterm premature rupture of membranes associated with intraamniotic infection. Infect Dis Clin North Am.

[14] Goncalves LF, Chaiworapongsa T, Romero R (2002). Intrauterine infection and prematurity. Ment Retard Dev Disabil Res Rev.

[15] Kim CJ, Romero R, Chaemsaithong P, Chaiyasit N, Yoon BH, Kim YM (2015). Acute chorioamnionitis and funisitis: definition, pathologic features, and clinical significance. Am J Obstet Gynecol.

[16] Jung E, Romero R, Yeo L, Diaz-Primera R, Marin-Concha J, Para R (2020). The fetal inflammatory response syndrome: the origins of a concept, pathophysiology, diagnosis, and obstetrical implications. Semin Fetal Neonatal Med.

[17] Gomez-Lopez N, Galaz J, Miller D, Farias-Jofre M, Liu Z, Arenas-Hernandez M (2022). The immunobiology of preterm labor and birth: intra-amniotic inflammation or breakdown of maternal-fetal homeostasis. Reproduction..

[18] Romero R, Kusanovic JP, Munoz H, Gomez R, Lamont RF, Yeo L (2010). Allergy-induced preterm labor after the ingestion of shellfish. J Matern Fetal Neonatal Med.

[19] D'Astous-Gauthier K, Graham F, Paradis L, Des Roches A, Begin P (2021). Beta-2 agonists may be superior to epinephrine to relieve severe anaphylactic uterine contractions. J Allergy Clin Immunol Pract.

[20] Tacquard C, Chassard D, Malinovsky JM, Saucedo M, Deneux-Tharaux C, Mertes PM (2019). Anaphylaxis-related mortality in the obstetrical setting: analysis of the French National Confidential Enquiry into Maternal Deaths from 2001 to 2012. Br J Anaesth.

[21] McCall SJ, Bonnet MP, Ayras O, Vandenberghe G, Gissler M, Zhang WH (2020). Anaphylaxis in pregnancy: a population-based multinational European study. Anaesthesia.

[22] Simionescu AA, Danciu BM, Stanescu AMA. Severe anaphylaxis in pregnancy: a systematic review of clinical presentation to determine outcomes. J Pers Med. 2021;11:1–13. 10.3390/jpm11111060 PMC8623240 34834412

[23] McCall SJ, Bunch KJ, Brocklehurst P, D'Arcy R, Hinshaw K, Kurinczuk JJ (2018). The incidence, characteristics, management and outcomes of anaphylaxis in pregnancy: a population-based descriptive study. BJOG.

[24] Cardona V, Ansotegui IJ, Ebisawa M, El-Gamal Y, Fernandez Rivas M, Fineman S (2020). World allergy organization anaphylaxis guidance 2020. World Allergy Organ J.

[25] Schultz WH. Physiological studies in anaphylaxis: the reaction of smooth muscle of the guinea pig sensitized with horse serum. J Pharmacol Exp Ther. 1910;1:549–67.

[26] Dale H. The anaphylactic reaction of plain muscle in the guinea-pig. J Pharmacol Exp Ther. 1913:167–223.

[27] Chand N, Eyre P (1978). The Schultz-Dale reaction: a review. Agents Actions.

[28] Kendall Alexander, HL, Holmes, JA. The effects of formaldehyde on smooth muscle contraction in anaphylaxis: studies in bacterial metabolism. J Inf Dis. 1927:137–42.

[29] Schild HO (1936). Reaction of the guinea-pig's uterus immersed in a histamine solution to histamine and anaphylaxis. J Physiol.

[30] Schild HO (1939). Histamine release in anaphylactic shock from various tissues of the guinea-pig. J Physiol..

[31] Kulka AM (1943). Studies on antibody antigen mixtures. II. The effect on normal living excised tissue and its dependence on the presence of free antibody in the mixture. J Immun..

[32] Kabat EA, Coffin GS, Smith DJ (1947). A quantitative study of passive anaphylaxis in the guinea pig. J Immunol.

[33] Swineford O Jr, Reynolds RJ. Studies in bacterial allergy. IV. The transitory nature of desensitization of the passively sensitized guinea pig uterus with a bacterial hapten, with a note on 95 per cent O_2_-5 per cent CO_2_ as an aerating mixture. J Allergy. 1951;22:156–9. 10.1016/0021-8707(51)90054-8 14823827

[34] Mongar JL, Schild HO (1952). A comparison of the effects of anaphylactic shock and of chemical histamine releasers. J Physiol.

[35] Makari JG (1955). Detection of soluble carcinoma antigen by use of the Schultz-Dale test. Am J Pathol.

[36] Makari JG (1955). Use of Schultz-Dale test for detection of specific antigen in sera of patients with carcinoma. Br Med J.

[37] Mongar JL, Schild HO (1957). Inhibition of the anaphylactic reaction. J Physiol.

[38] Sanyal RK, West GB (1957). 5-Hydroxytryptamine and anaphylactic shock. Nature.

[39] Burrows D (1958). Schultz-Dale test for detection of specific antigen in sera of patients with carcinoma. Br Med J.

[40] Fink MA, Gardner CE (1958). Anaphylaxis in guinea pig: improbability of release of serotonin in the Schultz-Dale reaction. Proc Soc Exp Biol Med.

[41] McEwen LM (1959). The Schultz-Dale anaphylactic test for carcinoma antigen. Br Med J..

[42] Mota I (1959). Effect of antigen and octylamine on mast cells and histamine content of sensitized guinea-pig tissues. J Physiol.

[43] Boreus LO (1961). Quantitative differences between guinea-pig ileum and uterus in the Schultz-Dale reaction. Acta Physiol Scand.

[44] Boreus LO, Westerholm B (1962). 5-Hydroxytryptamine in the Schultz-Dale reaction. Acta Physiol Scand.

[45] Dale MM (1965). The applicability of anaphylactic tests in studies of antigen mixtures. II. The discriminatory capacity of the tests. Immunology.

[46] Aronson AS (1968). The Schultz-Dale reaction of the depolarized guinea-pig uterus. Br J Pharmacol.

[47] Kellaway CH (1930). The anaphylactic reaction of the isolated uterus of the rat. Br J Exp Pathol..

[48] Suden CT (1934). Reactions of rat uterus excised and in situ to histamine and anaphylaxis. Am J Physiol.

[49] Sanyal RK, West GB (1958). Anaphylactic shock in the albino rat. J Physiol..

[50] Fink MA, Rothlauf MV (1955). In vitro anaphylaxis in the sensitized mouse uterus. Proc Soc Exp Biol Med.

[51] Fink MA (1956). Anaphylaxis in the mouse: possible relation of the Schultz-Dale reaction to serotonin release. Proc Soc Exp Biol Med.

[52] Tollackson KA, Frick OL (1966). Response of human smooth muscle in Schultz-Dale experiments. J Allergy.

[53] Garfield RE, Irani AM, Schwartz LB, Bytautiene E, Romero R (2006). Structural and functional comparison of mast cells in the pregnant versus nonpregnant human uterus. Am J Obstet Gynecol.

[54] Rudolph MI, Reinicke K, Cruz MA, Gallardo V, Gonzalez C, Bardisa L (1993). Distribution of mast cells and the effect of their mediators on contractility in human myometrium. Br J Obstet Gynaecol.

[55] Padilla L, Reinicke K, Montesino H, Villena F, Asencio H, Cruz M (1990). Histamine content and mast cells distribution in mouse uterus: the effect of sexual hormones, gestation and labor. Cell Mol Biol.

[56] Garfield RE, Bytautiene E, Vedernikov YP, Marshall JS, Romero R (2000). Modulation of rat uterine contractility by mast cells and their mediators. Am J Obstet Gynecol.

[57] Bytautiene E, Vedernikov YP, Saade GR, Romero R, Garfield RE (2002). Endogenous mast cell degranulation modulates cervical contractility in the guinea pig. Am J Obstet Gynecol.

[58] Bytautiene E, Vedernikov YP, Saade GR, Romero R, Garfield RE (2008). IgE-independent mast cell activation augments contractility of nonpregnant and pregnant guinea pig myometrium. Int Arch Allergy Immunol.

[59] Cruz MA, Gonzalez C, Acevedo CG, Sepulveda WH, Rudolph MI (1989). Effects of histamine and serotonin on the contractility of isolated pregnant and nonpregnant human myometrium. Gynecol Obstet Invest.

[60] Bytautiene E, Vedernikov YP, Saade GR, Romero R, Garfield RE (2003). Effect of histamine on phasic and tonic contractions of isolated uterine tissue from pregnant women. Am J Obstet Gynecol.

[61] Bytautiene E, Romero R, Vedernikov YP, El-Zeky F, Saade GR, Garfield RE (2004). Induction of premature labor and delivery by allergic reaction and prevention by histamine H1 receptor antagonist. Am J Obstet Gynecol.

[62] Willets JM, Taylor AH, Shaw H, Konje JC, Challiss RA (2008). Selective regulation of H1 histamine receptor signaling by G protein-coupled receptor kinase 2 in uterine smooth muscle cells. Mol Endocrinol.

[63] Romero R, Kusanovic JP, Gomez R, Lamont R, Bytautiene E, Garfield RE (2010). The clinical significance of eosinophils in the amniotic fluid in preterm labor. J Matern Fetal Neonatal Med.

[64] Martin LB, Kita H, Leiferman KM, Gleich GJ (1996). Eosinophils in allergy: role in disease, degranulation, and cytokines. Int Arch Allergy Immunol.

[65] Fulkerson PC, Rothenberg ME (2013). Targeting eosinophils in allergy, inflammation and beyond. Nat Rev Drug Discov.

[66] Wechsler ME, Munitz A, Ackerman SJ, Drake MG, Jackson DJ, Wardlaw AJ (2021). Eosinophils in health and disease: a state-of-the-art review. Mayo Clin Proc.

[67] Dunn JLM, Rothenberg ME (2022). 2021 year in review: spotlight on eosinophils. J Allergy Clin Immunol.

[68] Lombardi C, Berti A, Cottini M (2022). The emerging roles of eosinophils: implications for the targeted treatment of eosinophilic-associated inflammatory conditions. Curr Res Immunol.

[69] Kramer MS, Coates AL, Michoud MC, Dagenais S, Moshonas D, Davis GM (1995). Maternal asthma and idiopathic preterm labor. Am J Epidemiol.

[70] Liu S, Wen SW, Demissie K, Marcoux S, Kramer MS (2001). Maternal asthma and pregnancy outcomes: a retrospective cohort study. Am J Obstet Gynecol.

[71] Kojima R, Yokomichi H, Akiyama Y, Ooka T, Miyake K, Horiuchi S (2021). Association between preterm birth and maternal allergy considering IgE level. Pediatr Int.

[72] Klein VR, Harris AP, Abraham RA, Niebyl JR (1984). Fetal distress during a maternal systemic allergic reaction. Obstet Gynecol.

[73] Habek D, Cerkez-Habek J, Jalsovec D (2000). Anaphylactic shock in response to wasp sting in pregnancy. Zentralbl Gynakol.

[74] Shingai Y, Nakagawa K, Kato T, Fujioka T, Matsumoto T, Kihana T (2002). Severe allergy in a pregnant woman after vaginal examination with a latex glove. Gynecol Obstet Invest.

[75] Tsuzuki Y, Narita M, Nawa M, Nakagawa U, Wakai T (2017). Management of maternal anaphylaxis in pregnancy: a case report. Acute Med Surg.

[76] Donahue JG, Lupton JB, Golichowski AM (1995). Cutaneous mastocytosis complicating pregnancy. Obstet Gynecol.

[77] Kehoe SL, Bathgate SL, Macri CJ (2006). Use of a doula for labor coaching in a patient with indolent systemic mastocytosis in pregnancy. Obstet Gynecol.

[78] Madendag IC, Madendag Y, Tarhan I, Altinkaya SO, Danisman N (2010). Mastocytosis in pregnancy. Taiwan J Obstet Gynecol.

